# A Test of Double Interspecific Introgression of Nucleoporin Genes in *Drosophila*

**DOI:** 10.1534/g3.114.014027

**Published:** 2014-08-28

**Authors:** Kyoichi Sawamura, Kazunori Maehara, Yoko Keira, Hiroyuki O. Ishikawa, Takeshi Sasamura, Tomoko Yamakawa, Kenji Matsuno

**Affiliations:** * Faculty of Life and Environmental Sciences, University of Tsukuba, Tsukuba, Ibaraki 305-8572; † Graduate School of Life and Environmental Sciences, University of Tsukuba, Tsukuba, Ibaraki 305-8572; ‡ Department of Biology, Chiba University, Chiba, Chiba 263-8522; § Department of Biological Sciences, Osaka University, Toyonaka, Osaka, Japan 560-0043

**Keywords:** *Drosophila*, hybrid inviability, hybrid sterility, nucleoporin, reproductive isolation, speciation

## Abstract

In interspecific hybrids between *Drosophila melanogaster* and *Drosophila simulans*, the *D. simulans* nucleoporin-encoding *Nup96^sim^* and *Nup160^sim^* can cause recessive lethality if the hybrid does not also inherit the *D. simulans* X chromosome. In addition, *Nup160^sim^* leads to recessive female sterility in the *D. melanogaster* genetic background. Here, we conducted carefully controlled crosses to better understand

the relationship between *Nup96^sim^* and *Nup160^sim^*. *Nup96^sim^* did not lead to female sterility in the *D. melanogaster* genetic background, and double introgression of *Nup96^sim^* and *Nup160^sim^* did not generally lead to lethality when one was heterozygous and the other homozygous (hemizygous). It appears that introgression of additional autosomal *D. simulans* genes is necessary to cause lethality and that the effect of the introgression is dominant to *D. melanogaster* alleles. Interestingly, the genetic background affected dominance of *Nup96^sim^*, and double introgression carrying homozygous *Nup96^sim^* and hemizygous *Nup160^sim^* resulted in lethality. Thus, *Nup96^sim^* and *Nup160^sim^* seem to be two components of the same incompatibility.

A handful of hybrid incompatibility genes that are responsible for reproductive isolation between species have been identified (Johnson 2010; Presgraves 2010; Maheshwari and Barbash 2011; Ferree and Prasad 2012; Sawamura 2012). Surprisingly, two of these genes in the genus *Drosophila* encode the nuclear pore proteins (nucleoporins = Nups), which were previously thought to be functionally conserved among diverse organisms. Approximately 30 different Nups assemble to form the nuclear pore complex (NPC) and are essential for nucleocytoplasmic transport, gene regulation, and kinetochore formation (Bapteste *et al.* 2005; Strambio-De-Castillia *et al.* 2010; Adams and Wente 2013). *Nup96* and *Nup160* have been identified as reproductive isolation genes by deficiency mapping in which male hybrids were rescued from the independent lethality by *Lethal hybrid rescue* (*Lhr*) mutation of *D. simulans*. *D. melanogaster*/*D. simulans* hybrids carrying the *D. simulans Nup96^sim^* and *Nup160^sim^* are lethal in hemizygotes (or homozygotes) if they do not inherit the *D. simulans* X chromosome (Figure 1, A and B), and *Nup160^sim^* leads to recessive female sterility in the *D. melanogaster* genetic background (Presgraves *et al.* 2003; Tang and Presgraves 2009; Sawamura *et al.* 2010). Furthermore, positive natural selection and intermolecular coevolution have been demonstrated for several Nup genes including *Nup96* and *Nup160* in the genus *Drosophila* (Presgraves and Stephan 2007; Clark and Aquadro 2010; Mensch *et al.* 2013; Nolte *et al.* 2013).

**Figure 1:**
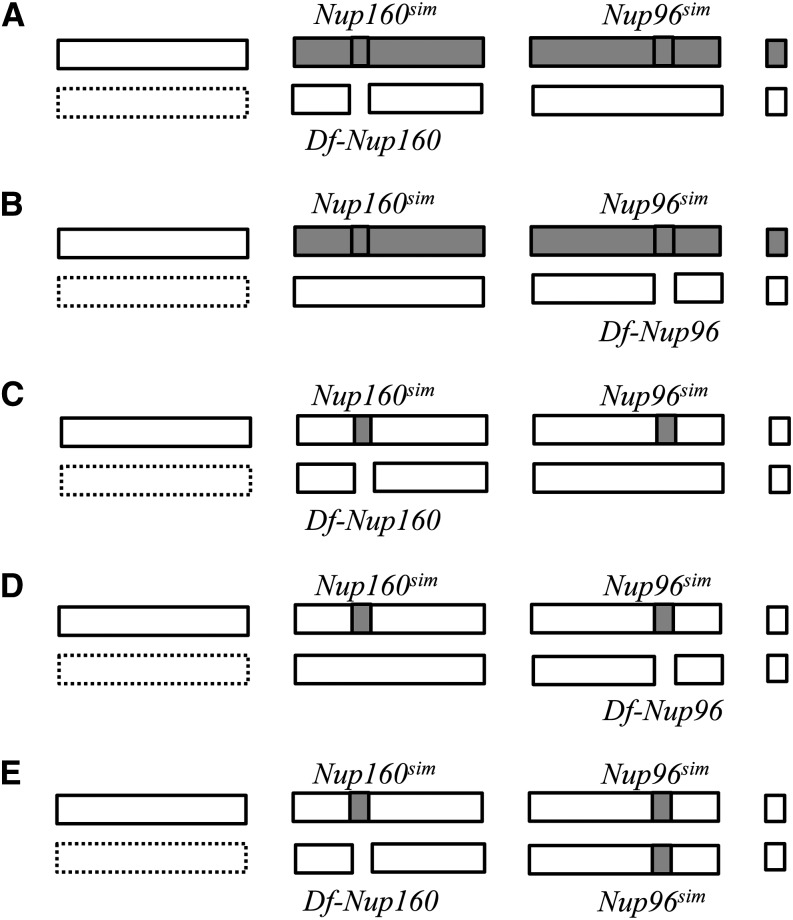
Genotypes examined previously and in this study. Pairs of bars represent chromosomes X, 2, 3, and 4 (left to right). Open bars (dashed if the presence is not obligate) indicate chromosomes/regions from *D. melanogaster*, and gray bars indicate chromosomes/regions from *D. simulans*. *D. simulans* alleles of *Nup160* and *Nup96* and the deficiencies on *D. melanogaster* chromosomes are also indicated. (A) Flies of this genotype all die according to Tang and Presgraves (2009) and Sawamura *et al.* (2010). (B) Flies of this genotype all die according to Presgraves *et al.* (2003). (C, D) These flies are viable according to the present analysis. (E) Flies of this genotype all die according to the present analysis. The genotypes in (A) and (B) are usually males carrying one X chromosome from *D. melanogaster*, but females carrying two *D. melanogaster* X chromosomes can also be obtained using the attached-X system (Presgraves *et al*., 2003; Tang & Presgraves 2009). The genotypes in (C), (D), and (E) are females carrying two *D. melanogaster* X chromosomes or males carrying one *D. melanogaster* X chromosome.

Both Nup96 and Nup160 (yeast homologs are Nup145C and Nup120, respectively) are components of the conserved Nup107−160 complex that has a role in the initial assembly of the NPC and functions as a stable anchoring point for other Nups—referred to as central scaffold Nups (Walther *et al.* 2003; Rasala *et al.* 2006; Grossman *et al.* 2012). The Nup107−160 complex forms a Y-shaped structure composed of two short arms—one composed of Nup160 and the other of Nup85—and an extended stalk that is connected to the two arms by Nup96 (Lutzmann *et al.* 2002; Brohawn *et al.* 2008; Bilokapic and Schwartz 2012; Szymborska *et al.* 2013). Because Nup96 and Nup160 interact directly (Leducq *et al.* 2012), it is reasonable to speculate that the lethality caused by *Nup96^sim^* and that caused by *Nup160^sim^* in the *D. melanogaster/D. simulans* hybrids are two distinct aspects of the same incompatibility. In this context, it is notable that protein−protein interactions between Nup96 and Nup160 are species-specific, as revealed in yeast sibling species and their hybrids (Leducq *et al.* 2012).

We conducted interspecific crosses of *Drosophila* to address the following three questions. (1) Does *Nup96^sim^* lead to female sterility in the *D. melanogaster* genetic background as seen with *Nup160^sim^* introgression? (2) Does the *Nup96^sim^* and *Nup160^sim^* double introgression lead to lethality when one is heterozygous and the other homozygous (or hemizygous) in the *D. melanogaster* background (Figure 1, C and D)? (3) Does the *Nup96^sim^* and *Nup160^sim^* double introgression lead to lethality when both are homozygous (or hemizygous) in the *D. melanogaster* background (Figure 1E)? Based on these three tests, we ask whether the double introgression of *Nup96^sim^* and *Nup160^sim^* is necessary and sufficient condition for the incompatibility to the gene(s) on the *D. melanogastere* X chromosome. Dominance of the genes and the possible involvement of different genes to the hybrid lethality will also be discussed.

## Materials and Methods

A genomic fragment of ~20.9 kb, including three open reading frames (*CG10208*, *Nup98-96*, and *mbc*), was amplified from DSM1-010P23, a *D. simulans* bacterial artificial chromosome clone established by the National BioResource Project Drosophila (Murakami *et al.* 2008), by polymerase chain reaction using the primers LA-AscI-F (5′-AGGCGCGCCTTACTTGCGACGGAACACCTCGACCTTGAG-3′), LA-*Bam*HI-R (5′-CGCGGATCCACGCACCTGGACAATGCAAGAGGGTGATTTG-3′), RA-*Bam*HI-F (5′-CGCGGATCCGACCAGCATGAGCATTGCCAACAGCATGCT-3′), and RA-*Pac*I-R (5′-ACCTTAATTAATCAGCACACCGGGCATAAGGTATCCCTGCTC-3′). This fragment was subcloned into the vector *attB*-P[acman]-Cm^R^ by homologous recombination (Venken *et al.* 2006). The construct was injected into embryos from *D. melanogaster* strain *y sc v P{y^+t7.7^ = nos-phiC31\int.NSL}X*; *P{y^+t7.7^ = CaryP}attP2* to allow for φC31-targeted, site-specific recombination into the *attP* landing site (cytological position 68A4 on chromosome 3) (Groth *et al.* 2004; Bateman *et al.* 2006; Bischof *et al.* 2007). The resultant transgene is abbreviated as *P{w^+^ Nup96^sim^}* in the present report.

**Figure 2:**
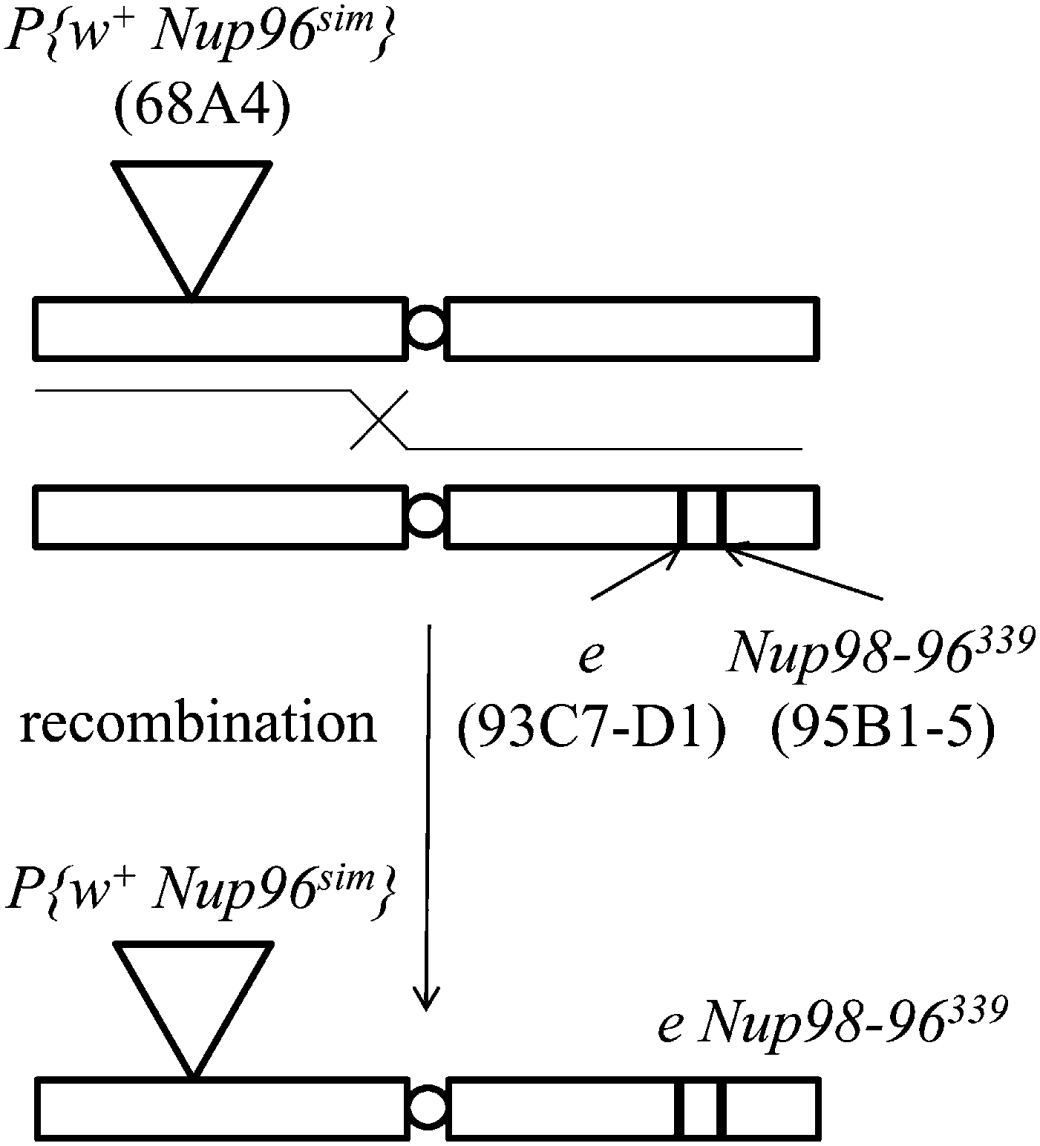
Construction of chromosome *P{w^+^ Nup96^sim^} e Nup98-96^339^* in the X-linked *w* mutant background. The *w^+^ e* recombinant (potentially *P{w^+^ Nup96^sim^} e Nup98-96^339^*) is produced by crossing *P{w^+^ Nup96^sim^}* and *e Nup98-96^339^*.

A *P{w^+^ Nup96^sim^} e Nup98-96^339^* chromosome was made by recombination between *P{w^+^ Nup96^sim^}* and *e Nup98-96^339^* chromosomes in the *w* genetic background (Figure 2). Here *w^+^* (68A4; red eye color) and *e* (93C7-D1; ebony/dark body color) were used as visible markers, and *Nup98-96* is at 95B1-5. To confirm that the recombinant chromosome carried the *Nup98-96^339^* mutation and that it was not lost by rare double recombination between *e* and *Nup98-96^339^*, *P{w^+^ Nup96^sim^}* was removed from the established chromosome by further recombination with a wild-type chromosome using the *w^+^* and *e* markers. The resultant chromosome again exhibited recessive lethality that was not complemented by the *Nup98-96* deficiencies (*Df(3R)Exel9014* and *Df(3R)BSC489*), thus confirming that the chromosome examined carried *Nup98-96^339^*. A balancer chromosome, *TM3*, was used to isolate the recombinant chromosome in a heterozygous state, and *CyO* and *SM1* were used as a chromosome 2 balancer. *Int(2L)D+S* is a chromosome 2 *D. simulans* introgression covering two cytological regions that include *Nup160^sim^* (Sawamura *et al.* 2000). Of note, the *Int(2L)D+S* introgression also carries other *Nup* loci (*Nup107* and *Nup154*), but we do not believe that this could affect our overall conclusion of this study. When necessary, only *Nup160^sim^* was made hemizygous by a deficiency of the *Nup160* locus, *Df(2L)Nup160M190* (Maehara *et al.* 2012).

## Results

First, we established a *D. melanogaster* line carrying an extra segment of *D. simulans* chromosome 3 (including *CG10208*, *Nup98-96*, and *mbc*) inserted at cytological position 68A4 of the same chromosome. Note that *Nup98-96* is a dicistronic gene that produces the proteins Nup98 and Nup96 by autoproteolysis (Presgraves *et al.* 2003). Then, the endogenous *Nup98-96* at 95B1-5 of the line was replaced by the recessive lethal *Nup98-96^339^* mutant allele (Figure 2), which has a stop codon at amino acid position 1726 (therefore, only *Nup96* was affected; Presgraves *et al.* 2003). Thus, we obtained a *D. melanogaster* chromosome 3 carrying *Nup96^sim^* instead of the *D. melanogaster* wild-type allele of *Nup96*. The resultant chromosome (*P{w^+^ Nup96^sim^} e Nup98-96^339^*) is referred to as the *Nup96^sim^* introgression. Both male and female *Nup96^sim^* introgression homozygotes (and hemizygotes) were viable and fertile, and the strain homozygous for *Nup96^sim^* could be maintained indefinitely. Although females that were homozygous for *Nup96^sim^* and hemizygous over *Df(3R)BSC489* exhibited lower fertility than heterozygous controls (χ^2^ = 94.5, *P* < 0.001 and χ^2^ = 6.6576, *P* < 0.05, respectively), fertility was not decreased in *Nup96^sim^* hemizygotes over *Df(3R)Exel9014* (χ^2^ = 1.5958, *P* > 0.2) (Table 1). Therefore, *Nup96^sim^* does not lead to female sterility in the *D. melanogaster* genetic background. We note the possibility that the chromosome harboring *Nup96^sim^* might have a second-site recessive gene or genes responsible for lower female fertility.

**Table 1:** Hatchability of eggs from females crossed with wild-type *D. melanogaster* males

Maternal Genotype*^a^*	Number of Eggs		Hatchability, %
	Collected	Hatched	
*Nup96^sim^* heterozygotes over *TM3*	200	191	95.5
*Nup96^sim^* homozygotes	200	106	53.0
*Nup96^sim^* hemizygotes over *Df(3R)Exel9014*	200	185	92.5
*Nup96^sim^* hemizygotes over *Df(3R)BSC489*	200	177	88.5

a The full genotype of *Nup96^sim^* is *P{w^+^ Nup96^sim^} e Nup98-96^339^*.

Next, to examine possible synergistic and/or additive effects of *Nup160^sim^* and *Nup96^sim^* introgression, we produced *w*; *Int(2L)D+S*, *Nup160^sim^/CyO*; *Nup96^sim^ e/+* males by conventional crosses. Then, these males were crossed to females heterozygous for a balancer and a mutation (or a deficiency) of *Nup160* or *Nup98-96*. If the introgressions were behaving similar to the F_1_ hybrid, then *Nup160^sim^/*(*Nup160^sim^* or *Df-Nup160*); *Nup96^sim^/+* is expected to be lethal; however, that is not what is observed. Instead, the *Nup160^sim^* homozygotes (or hemizygotes) were viable in the *Nup96^sim^* heterozygous background (Figure 1C and Table 2). If the introgressions were behaving similar to the F_1_ hybrid, then *Nup160^sim^/+*; *Nup96^sim^/*(*Nup96^sim^* or *Df-Nup96*) is expected to be lethal; however, that is not what is observed. Instead, the *Nup96^sim^* homozygotes (or hemizygotes) were viable in the *Nup160^sim^* heterozygous background (Figure 1D and Table 3). Thus, the *Nup96^sim^* and *Nup160^sim^* double introgression did not lead to lethality when one was heterozygous and the other homozygous (or hemizygous).

**Table 2:** Viability of flies homozygous (or hemizygous) for *Nup160^sim^* and heterozygous for *Nup96^sim^*

Maternal genotype*^a^*	Number of Flies			
	Cy w	Cy w^+^	Cy^+^ w	Cy^+^ w^+^ (Viability*^b^*)
				
*w*; *Int(2L)D+S*, *Nup160^sim^/CyO*				
Genotype	*Nup160^sim^/*+; +/+	*Nup160^sim^/*+; *Nup96^sim^/*+	*Nup160^sim^/Nup160^sim^*; +/+	*Nup160^sim^/Nup160^sim^*; *Nup96^sim^/*+
Females	132	202	35*^c^*	25 (0.71)*^c^*
Males	146	206	39*^c^*	35 (0.90)*^c^*
* w*; *Df(2L)Nup160M190/CyO*				
Genotype	*(Nup160^sim^* or *Df-Nup160)/*+; +/+	*(Nup160^sim^* or *Df-Nup160)/*+; *Nup96^sim^/*+	*Nup160^sim^/Df-Nup160*; +/+	*Nup160^sim^/Df-Nup160*; *Nup96^sim^/*+
Females	180	201	77	20 (0.26)
Males	155	188	105	68 (0.65)
Segregation ratio expected	2	2	1	1

a Crossed with *w*; *Int(2L)D*+*S*, *Nup160^sim^/CyO*; *Nup96^sim^/*+ males. The balancer *CyO* has *Cy* as a dominant marker.

b Calculated as (number of flies in the fourth class) divided by (number of flies in the third class).

c The viability of *Int(2L)D*+*S* homozygotes was low because of linked recessive lethals that presumably accumulated on the chromosome.

**Table 3:** Viability of flies heterozygous for *Nup160^sim^* and hemizygous for *Nup96^sim^*

Maternal genotype*^b^*	Number of Flies							
	Cy w Sb	Cy w Sb^+^	Cy w^+^ Sb	Cy w^+^ Sb^+^	Cy^+^ w Sb	Cy^+^ w Sb^+^	Cy^+^ w^+^ Sb	Cy^+^ w^+^ Sb^+^ (Viability*^a^*)
								
* w*; *Nup98-96^339^/TM3*								
Genotype	+/+; +/+	+/+; *l(3)Nup96/*+	+/+; *Nup96^sim^/*+	+/+; *Nup96^sim^/l(3)Nup96*	*Nup160^sim^/*+; +/+	*Nup160^sim^/*+; *l(3)Nup96/*+	*Nup160^sim^/*+; *Nup96^sim^/*+	*Nup160^sim^/*+; *Nup96^sim^/l(3)Nup96*
Females	38	123	50	84	72	102	84	72 (0.86)
Males	61	89	70	62	87	109	90	69 (1.11)
* w*; *Df(3R)BSC489/TM6C*								
Genotype	+/+; +/+	+/+; *Df-Nup96/*+	+/+; *Nup96^sim^/*+	+/+; *Nup96^sim^/Df-Nup96*	*Nup160^sim^/*+; +/+	*Nup160^sim^/*+; *Df-Nup96/*+	*Nup160^sim^/*+; *Nup96^sim^/*+	*Nup160^sim^/*+; *Nup96^sim^/Df-Nup96*
Females	123	170	63	142	98	151	62	92 (0.65)
Males	117	135	28	106	85	128	76	65 (0.61)
Segregation ratio expected	1	1	1	1	1	1	1	1

a Calculated as (number of flies in the eighth class) divided by (number of flies in the fourth class).

b They were crossed to *w*; *Int(2L)D*+*S*, *Nup160^sim^/CyO*; *Nup96^sim^/*+ males. The balancers *TM3* and *TM6C* have *Sb* (and *Ser* in the former) as a dominant marker. *l(3)Nup96* stands for a recessive mutation of the *Nup96* gene, *Nup98-96^339^*.

Finally, we attempted to make a strain carrying both *Nup160^sim^* and *Nup96^sim^* introgressions maintained with chromosome 2 and 3 balancers but were not successful, presumably because *Int(2L)D+S* can cause dominant male semisterility in some genetic backgrounds (S. Parhad, personal communication). Therefore, we could not test the viability/fertility of *Nup96^sim^* and *Nup160^sim^* double introgression homozygotes. Instead, we made *w*; *Df(2L)Nup160M190/SM1*; *Nup96^sim^/TM3* females and *w*; *Int(2L)D+S*, *Nup160^sim^/SM1*; *Nup96^sim^/+* males by conventional crosses and crossed them. *Int(2L)D+S*, *Nup160^sim^/ Df(2L)Nup160M190*; *Nup96^sim^/+* flies were viable as we previously noted (Table 2), although hemizygosity of *Nup160^sim^* might have reduced their viability (Table 4). Unexpectedly, we found that *Int(2L)D+S*, *Nup160^sim^/ Df(2L)Nup160M190*; *Nup96^sim^/TM3* was semilethal (Table 4). This suggests that dominance of *Nup96^sim^* may be affected by the genetic background. Furthermore, we found that *Int(2L)D+S*, *Nup160^sim^/ Df(2L)Nup160M190*; *Nup96^sim^/Nup96^sim^* was also absolutely lethal (Table 4 and Figure 1E). Thus, the protein products of *Nup96^sim^* and *Nup160^sim^* seem to interact directly.

**Table 4:** Viability of flies hemizygous for *Nup160^sim^* and homozygous for *Nup96^sim^*

Maternal genotype*^c^*	Number of Flies*^a^*							
	Cy w Sb	Cy w^+^ Sb^+^	Cy w^+^ Sb	Cy w^++^ Sb^+^	Cy^+^ w Sb	Cy^+^ w^+^ Sb^+^	Cy^+^ w^+^ Sb (Viability*^b^*)	Cy^+^ w^++^ Sb^+^ (Viability*^b^*)
								
*w*; *Df(2L)Nup160M190/SM1*; *Nup96^sim^/TM3*								
Genotype	(*Nup160^sim^* or *Df-Nup160*)/+; +/+	(*Nup160^sim^* or *Df-Nup160*)/+; *Nup96^sim^*/+	(*Nup160^sim^* or *Df-Nup160*)/+; *Nup96^sim^*/+ (*TM3*)	(*Nup160^sim^* or *Df-Nup160*)/+; *Nup96^sim^*/*Nup96^sim^*	*Nup160^sim^*/*Df-Nup160*; +/+	*Nup160^sim^*/*Df-Nup160*; *Nup96^sim^*/+	*Nup160^sim^*/*Df-Nup160*; *Nup96^sim^*/+ (*TM3*)	*Nup160^sim^*/*Df-Nup160*; *Nup96^sim^Nup96^sim^*
Females	436*^d^*	533	423	163	137	98*^e^*	1 (0.01)	0 (0)
Males	442*^d^*	547	452	190	145	177	8 (0.05)	0 (0)
Segregation ratio expected	2	2	2	2	1	1	1	1

a w^++^ means flies carrying two w^+^ markers; distinguished by their darker eye color. A few flies ambiguous for the Cy phenotype were excluded.

b Calculated as (number of flies in the seventh or eighth class) divided by (number of flies in the sixth class).

c Crossed with *w*; *Int(2L)D*+*S*, *Nup160^sim^/SM1*; *Nup96^sim^/*+ males. The balancers *SM1* and *TM3* have *Cy* and *Sb* (and *Ser*) as dominant markers, respectively.

d One was ebony presumably caused by a rare male recombination or a spontaneous mutation.

e One was a gynandromorph.

## Discussion

We found that *D. melanogaster* females homozygous (or hemizygous) for the *Nup96^sim^* introgression were fertile (Table 1), in contrast to what has been observed for the *Nup160^sim^* introgression, for which eggs produced by homozygotes (or hemizygotes) display karyogamy failure and female pronuclei never fuse to wild-type male pronuclei (Sawamura *et al.* 2004). Although Nup96 and Nup160 are functionally and structurally in close proximity in the Y-shaped Nup107−160 complex, the effects of interspecific substitution of these two components differed. The structural position of Nup96 and Nup160 might reflect the functional difference; Nup160 is on the surface of the pore ring (Bilokapic and Schwartz 2012; Szymborska *et al.* 2013) and might have more interactions with other proteins important for NPC function.

We found that flies with genotypes indicated in Figure 1, C and D were viable (Table 2 and Table 3), in contrast to the lethality observed for those with genotypes indicated in Figure 1, A and B (Presgraves *et al.* 2003; Tang and Presgraves 2009; Sawamura *et al.* 2010). The primary difference between these flies is the genetic background, with the remaining autosomal genes being from *D. melanogaster* in our flies and from *D. melanogaster* and *D. simulans* (heterozygous) in the previous studies. Apparently the presence of additional autosomal *D. simulans* genes is necessary to cause lethality, and these genes are dominant to the *D. melanogaster* alleles. Thus, more genes (maybe encoding other Nups) are involved in this hybrid incompatibility. *Nup107* and *Nup154* are excluded from the candidates because *Int(2L)D+S* also carries these genes from *D. simulans* but did not exhibit the dominant effect. One candidate for the interactant is *Nup75*, presumably the *Drosophila* homolog of *Nup85*. Further investigation of this system is necessary to better understand the genetic mechanisms of reproductive isolation.

Interestingly, dominance of *Nup96^sim^* was changed by the presence of a balancer *TM3* (Table 4). Reproductive isolation might be easily affected by the genetic background, as has been suggested in the other hybrid incompatibility (*Lhr vs. Hmr*) in the same species cross (Matute *et al.* 2014; Shirata *et al.* 2014). Finally, double introgression carrying homozygous *Nup96^sim^* and hemizygous *Nup160^sim^* resulted in lethality in the hybrids (Table 4 and Figure 1E). This is the first evidence suggesting that *Nup96^sim^* and *Nup160^sim^* are two components of the same incompatibility.
